# Molecular Dynamics Simulations of Nucleosomes Containing Histone Variant H2A.J

**DOI:** 10.3390/ijms252212136

**Published:** 2024-11-12

**Authors:** Nikita A. Kosarim, Anastasiia S. Fedulova, Aleksandra S. Shariafetdinova, Grigoriy A. Armeev, Alexey K. Shaytan

**Affiliations:** 1 Department of Biology, Lomonosov Moscow State University, 119234 Moscow, Russia; n.kosarim@intbio.org (N.A.K.); a.kniazeva@intbio.org (A.S.F.); armeev@intbio.org (G.A.A.); 2 Institute of Gene Biology, 119334 Moscow, Russia

**Keywords:** nucleosomes, histone variants, H2A.J, histone modifications, histone tails, protein–DNA interactions, molecular modeling, molecular dynamics simulations

## Abstract

Histone proteins form the building blocks of chromatin—nucleosomes. Incorporation of alternative histone variants instead of the major (canonical) histones into nucleosomes is a key mechanism enabling epigenetic regulation of genome functioning. In humans, H2A.J is a constitutively expressed histone variant whose accumulation is associated with cell senescence, inflammatory gene expression, and certain cancers. It is sequence-wise very similar to the canonical H2A histones, and its effects on the nucleosome structure and dynamics remain elusive. This study employed all-atom molecular dynamics simulations to reveal atomistic mechanisms of structural and dynamical effects conferred by the incorporation of H2A.J into nucleosomes. We showed that the H2A.J C-terminal tail and its phosphorylated form have unique dynamics and interaction patterns with the DNA, which should affect DNA unwrapping and the availability of nucleosomes for interactions with other chromatin effectors. The dynamics of the L1-loop and the hydrogen bonding patterns inside the histone octamer were shown to be sensitive to single amino acid substitutions, potentially explaining the higher thermal stability of H2A.J nucleosomes. Taken together, our study demonstrated unique dynamical features of H2A.J-containing nucleosomes, which contribute to further understanding of the molecular mechanisms employed by H2A.J in regulating genome functioning.

## 1. Introduction

**Figure 1:**
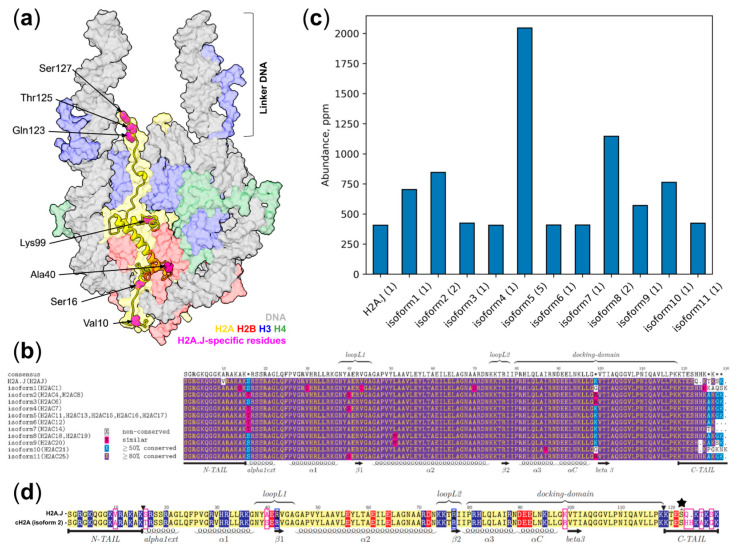
Nucleosomes containing the H2A.J histone variant, its sequence, and expression as compared to canonical histones: (**a**) A 3D model of the H2A.J-containing nucleosome. Histones and DNA are depicted in molecular surface representation using respective colors (see legend). Main residues specific to H2A.J (with respect to at least several canonical H2A isoforms) are colored in magenta and named; (**b**) Expression levels of H2A.J and canonical H2A isoforms as provided by PaxDB. Isoform number and corresponding human genes are given for each sequence; (**c**) Multiple sequence alignment of all canonical H2A isoforms and H2A.J histone in humans; (**d**) Alignment of H2A.J and isoform 2 of canonical H2A that was used in simulations. Positively and negatively charged residues are highlighted in blue and red, respectively. Key arginines that are inserted into DNA minor groves are highlighted with dark blue frames. The phosphorylation site of H2A.J is marked with a black asterisk. Magenta boxes mark H2A.J-specific residues.

Comprehensively understanding genome functioning and its regulation is a grand challenge for biology in the 21st century. Eukaryotic organisms rely on different epigenetic mechanisms for genome markup, regulation of gene expression, switching on and off various parts of the genome during cell differentiation, organisms’ development, accommodation to environmental conditions, and in response to diseases and aging. These epigenetic mechanisms include DNA methylation, post-translational modification (PTM) of histones, incorporation of histone variants, chromatin remodeling, utilization of different non-coding RNAs, etc. [1]. The 3D organization of the genome at different levels is integral to epigenetic regulation. At the very basic scale, eukaryotic genomes are compacted by histone proteins through the formation of nucleosomes [2]. In the nucleosome, a DNA segment is wrapped around the octamer of core histone proteins (H3, H4, H2A, H2B—two copies of each) in around 1.67 turns of a left-handed superhelix. The DNA stably associated with each histone octamer is around 147 bp in length and forms the nucleosome core particle (NCP), which is then connected to other NCPs through linker DNA segments (Figure 1a) [3]. Linker DNA segments may vary from around 10 to 90 base pairs in length depending on the type of organism, cell type, stage of the cell cycle, and location in the cell nucleus [4]. The linker histone H1 may additionally associate with the nucleosome to organize the linker DNA segments [5]. Nucleosomes not only compact genomic DNA but are the basic units of epigenetic regulation through harboring histone PTMs and histone variants and mediating the readout of these signatures either through direct interactions with effector domains of chromatin proteins or through affecting the nucleosome dynamics and stability [6].

The majority of histones, which are incorporated into nucleosomes during the S-phase of the cell cycle, are expressed from the so-called clustered (also called canonical) histone genes [7]. However, an important set of histone gene paralogs, called histone variants, are expressed during the cell cycle, often in a tissue-specific manner [8]. The incorporation of histone variants confers different properties to nucleosomes, altering their stability and providing new sites for interactions with chromatin proteins and sites for PTM. Histone variants are implicated in many genomic processes, including regulation of gene expression, maintenance of epigenetic states and their transmission during mitosis and potentially during meiosis, X-chromosome inactivation, DNA damage response, etc. [8,9]. Some histone variants, like H2A.Z, are present in all eukaryotes, and their knockouts are usually lethal [10]; others may be species-specific and involved in the delicate regulation of complex organism functions. For instance, the H2B.E histone variant in mice is implicated in the development of olfactory neurons [11]. Mutations of histone and deregulation of expression of their variants and isoforms are known to play a causative role in cancers or contribute to their development and progression [12]. H2A histones have the most number of variants. In the human genome, 17 genes are coding for clustered (canonical) H2A histones (many encoding identical protein products), and 12 genes are coding for H2A histone variants [13]. A peculiar feature of histone H2A is the presence of disordered tails on both C- and N-ends of the protein. While the major H2A histone variants, such as H2A.Z, H2A.X, and H2A.B, have been extensively characterized both biophysically and functionally, the characterization of other variants, especially those that are sequencewise similar to canonical histones and confer subtle structural and functional effects, has been lagging.

Among the less studied H2A variants is the H2A.J histone. In humans, it is encoded by the H2AJ gene located on chromosome 12; similar genes are present in other mammals [14]. In humans, H2AJ differs from the canonical histones only at several sequence positions (see Figure 1b); its gene is intronless, similar to canonical genes, but appears to produce only polyadenylated mRNA, while canonical genes lack poly-A tails in their mRNAs and use an alternative post-transcriptional regulation mechanism instead [15]. Canonical H2A histones in humans are expressed by 17 genes that encode 11 slightly different protein sequences (called canonical isoforms). H2A.J differs from all the canonical histones by an A10V substitution in the N-terminal tail and a different sequence of its C-terminal (up to the six terminal amino acid residues are different) (see Figure 1b). The most expressed canonical histone isoform (according to PaxDB; see Figure 1c) has a C-tail sequence SHHKAKGK, while H2A.J C-tail has the sequence SQKTKSK. Some minor canonical isoforms encode C-tails more similar to H2A.J, like SHKAKSK encoded by the H2AC20 gene. However, even in comparison to those isoforms, H2A.J C-tail differs by two substitutions—H123Q and A125T. The former leads to the presence of an SQ-motif in H2A.J—a potential minimal phosphorylation site that may be phosphorylated by ATM kinases, which are activated as a part of the DNA damage response (DDR) pathway [16]. This site was reported to be phosphorylated in mice at a low level after treatment with ionizing irradiation (1% of H2A.J was phosphorylated, compared to 50% of H2A.X) [17]. H2A.J, together with the majority of canonical histone isoforms, has alanine at position 40, while a subset of canonical H2As (encoded by H2AC4, H2AC8, H2AC7, and H2AC25) has serine at this position. Previous experimental analysis of the effects of the A40S substitution showed that substitution of A to S decreases the thermal stability of the nucleosomes, suggesting that H2A.J-containing nucleosomes are more stable than those encoded by certain canonical isoforms (see Figure 1b) [18]. The T16S and R99K substitutions that are found in H2A.J compared to some other canonical isoforms have been reported to not affect nucleosome stability [18].

H2A.J has been functionally characterized in humans and mice [14]. H2A.J was shown to accumulate in senescent human fibroblasts with persistent DNA damage and promote inflammatory gene expression [14,19]. In fibroblast irradiation experiments, it was shown that H2A.J colocalizes with 53BP1 and is incorporated at the periphery of the so-called senescence-associated chromatin foci (SAHF) [19]. Depletion of H2A.J via RNA interference has been shown to modify senescence-associated chromatin re-structuring and abolish the senescence-associated secretory phenotype in human fibroblasts when subjected to ionizing radiation [19]. The combination of immunofluorescence microscopy, ATAC-seq and RNA-seq analysis, ELISA and RT-PCR, as well as H2A.J-immunohistochemistry, demonstrated that differential incorporation of H2A.J has profound effects on higher-order chromatin organization and on establishing the epigenetic state of senescence. Thus, the overexpression of H2A.J is suggested to impede heterochromatin formation following IR exposure and therefore inhibit the SAHF-mediated gene silencing of proliferation-promoting genes. Thereby, H2A.J overexpression may potentially overcome senescence-associated growth arrest that serves as a potent anti-tumor mechanism by preventing the proliferation of potentially cancerous cells [20]. Functions and roles of H2A.J and all other mammalian H2A variants in cancer biology were recently reviewed by Lai and Chan, who concluded that H2A.J is overexpressed in a variety of cancers [21].

The mechanistic hypothesis explaining H2A.J’s association with inflammatory gene expression has been put forward by Mangelinck et al. [17]. They have demonstrated that H2A.J interacts with H1 histone weaker than canonical H2A (canonical H2A isoform 5 was used in the respective experiment, see Figure 1b), thus H2A.J contributes to weakening histone H1 association with chromatin. This weakening may promote the expression of repetitive DNA elements found in heterochromatin, which are known to trigger inflammatory responses. Dysregulation in H2A.J expression has also been found to be associated with some types of cancer and their resistance, including breast, brain, colorectal, and liver cancers [22,23,24,25,26]. Another interesting feature of H2A.J is its tissue and cell-type specificity. According to recent data from an immunohistochemical study (corroborated by GTEx data), expression of H2A.J is increased in luminal epithelial cells of some glands, in particular, mammary, prostate, pancreas, thyroid, salivary, and stomach tissues of mice and humans [27]. H2A.J accumulation in the liver and kidneys and senescent cells was also shown in another experimental study in mice (while GTEx data do not currently support the presence of such accumulation in human liver and kidneys) [14]. Interestingly, accumulation in senescent fibroblasts containing persistent DNA damage was not accompanied by higher H2A.J mRNA levels, suggesting that accumulation in senescence might involve a post-transcriptional regulation mechanism [14]. The functional significance of H2A.J-specific amino acid substitutions has been experimentally demonstrated by introducing substitutions either into H2A.J or canonical H2As. In particular, ectopic expression studies have suggested that the introduction of a V10A substitution into H2A.J (which makes the sequence more similar to canonical histones) results in an increased expression of inflammatory genes, suggesting that dimethyl groups of valine may form a part of a binding site for a regulatory factor that counteracts inflammatory gene expression [17]. In turn, the ectopic expression of H2A.J, whose tail was changed to that of a canonical H2A (isoform 5), unlike wild-type H2A.J, was ineffective at restoring inflammatory gene expression in cells with knock-downed H2A.J, suggesting that the C-tail of H2A.J is functionally important [14]. At the same time, the introduction of a S122E substitution into H2A.J mimicking phosphorylation was shown to lead to an even greater expression of inflammatory genes [17].

The structural basis of sequence-and-function relations is still elusive for many of the histone variants. Recently, a set of X-ray crystallography and cryo-electron microscopy structures with variant histones were obtained (reviewed in Kurumizaka et al. [28]; see also NucleosomeDB http://nucldb.intbio.org (accessed on 6 November 2024) [29]). These structural data provided variant-unique patterns of nucleosome structural features, for instance, increased or decreased NCP stability and flexibility of entry–exit DNA regions, or reorganization of the L1-L1-loop interactions between the two copies of H2A-histones (e.g., for the H2A.Z variant [30] and the macroH2A variant [31]). The only H2A.J-containing nucleosome structure was obtained in 2020 by Tanaka et al. using the X-ray diffraction method (PDB ID 6KVD) [18]. The study has shown that the H2A.J variant forms stable nucleosomes very similar to canonical ones. The H2A.J regions around the Ser16, Ala40, and Lys99 residues were not substantially changed, as compared to the canonical nucleosome structure.

Molecular dynamics simulations starting from the first trajectories obtained by Bishop et al. [32] and Ruscio and Onufriev [33] are becoming an important tool in analyzing nucleosome properties at the atomistic scale (reviewed recently by us in Fedulova et al. [34]). For example, using MD simulations, it was possible to characterize the reorganization of L1-loop regions in different histone variants, show that they play a pivotal role in stabilizing DNA binding to the octamer through direct interactions [35,36], characterize core structural rearrangements, their plasticity, and their role in DNA sliding [37], and analyze altered allosteric networks in the nucleosome [35]. The effects conferred by different histone variants were also characterized. For example, Kohestani et al. showed that H2A.B incorporation promotes nucleosome unwrapping due to the weakening of some intranucleosomal interactions [38], while Peng et al. found that the N-terminus of H2A.B may somewhat compensate for the unwrapping effect [39]. Li et al. demonstrated that both N- and C-terminal tails of H2A.Z play major roles in making nucleosomes more mobile and their DNA more accessible [40]. The effects of histone PTMs were also characterized [41]. Bui et al. demonstrated that for nucleosomes containing the CENP-A H3 histone variant, only one post-translational modification (acetylation of CENP-A Lys124) causes histone core tightening and hinders accessibility to its C-terminus, diminishing CENP-C binding [42]. Understanding of the nucleosome unwrapping process, histone tail dynamics, and development of optimal force fields for modeling are also active areas of ongoing research [43,44,45]. Altogether, MD simulations are helpful in providing mechanistic insights into the functioning of nucleosomes incorporating histone variants.

In the case of the H2A.J histone variant, there are no MD studies to date. In this work, using atomistic MD simulations, we have explored the structure and dynamics of human nucleosomes harboring the H2A.J histone variant and its phosphorylated form at Ser122. We demonstrated that although the overall dynamics of canonical and H2A.J-containing nucleosomes are similar, the sequence differences in the L1-loops and C-terminal tails confer unique dynamical properties and histone–histone and histone–DNA interaction patterns within nucleosomes. We discuss the implications of these differences on nucleosome functioning in the context of the known experimental data on H2A.J’s involvement in certain cellular processes.

## 2. Results

### 2.1. Overview of MD Simulations

**Figure 2:**
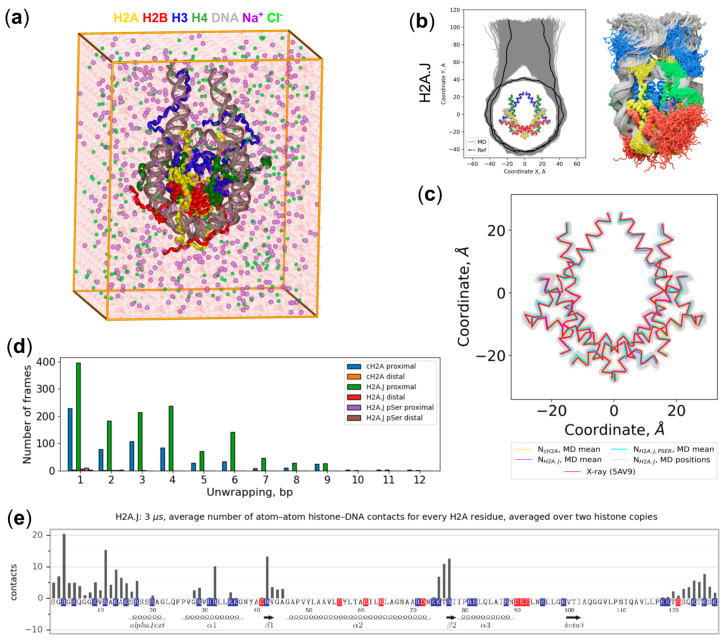
MD simulations overview: (**a**) The starting structure for simulations: nucleosome with linker DNA segments in a simulation box with solvent; (**b**) Right: MD snapshots overlay. Left: overlaid projections of the DNA base pair center positions and Cα-atoms’ positions of histones’ α2-helices onto the plane perpendicular to the nucleosomal superhelical axis (nucleosomal plane). Snapshots are spaced every 100 ns; (**c**) Average conformations of the histone α2-helices of different simulated systems and conformational fluctuations of the said helices in H2A.J-containing nucleosomes as visualized by the projections of Cα-atoms’ coordinates onto the nucleosomal plane; (**d**) Histogram showing the different unwrapping states of the nucleosomal DNA observed during simulations; (**e**) The profile of the average number of atom–atom contacts between H2A histone residues and DNA for the H2A.J-containing system (N_H2A.J_). See details in Supplementary Figure S4. Positively and negatively charged residues are highlighted in blue and red, respectively.

In this study, we constructed three atomistic models of human nucleosomes with full histone tails and 20 bp linker DNA segments: N_cH2A_ contains canonical H2A histones (cH2A, isoform 2 encoded by H2AC4 or H2AC8 genes); N_H2A.J_ contains native H2A.J histones (encoded by the H2AJ gene); N_H2A.J-pSer_ contains H2A.Js phosphorylated at Ser122. Each model was solvated in a rectangular box with explicit solvent and NaCl 150 mM concentration (Figure 2a), equilibrated, after which two production runs were performed (run 1 and run 2) for at least 1 microsecond using the all-atom MD method (for more details see the Materials and Methods section and Supplementary Table S1; see Figure 2b for the representation of the observed dynamics).

The overall analysis of nucleosome dynamics for the three systems is presented in Figure 2; interactive trajectory previews are available at http://intbio.org/Kosarim_et_al_2024/ (accessed on 6 November 2024). Visual inspection of the nucleosome dynamics, analysis of nucleosome conformations using 2D projections along the planes of the nucleosome reference frame, and DNA unwrapping analysis did not reveal any substantial statistically significant difference between the three simulated systems (Figure 2, Supplementary Figures S1 and S2; interactive trajectory preview is available at http://intbio.org/Kosarim_et_al_2024/ (accessed on 6 November 2024)). A comparison of the average conformation of the core histone α2-helices using their 2D projection onto the nucleosomal plane did not reveal any substantial differences between the simulated systems (Figure 2c). In all the simulations, we observed nucleosome breathing events (unwrapping of the nucleosomal DNA terminal helical turns), although their extent varied between the runs and depending on whether the left (proximal) or right (distal) side of the nucleosome was examined, concomitant with earlier observations that it is a stochastic process with equilibration times exceeding our trajectory length [37]. The considerable asymmetric core nucleosomal DNA unwrapping of up to 13 base pairs was observed in the simulated systems (Figure 2d, Supplementary Figure S3). Except for these DNA breathing/unwrapping motions, nucleosomes in all trajectories maintained stable structures, consistent with experimental observations that demonstrated the stability of H2A.J and canonical nucleosomes [18]. The profile of average atom–atom contacts between the residues of H2A.J and the DNA is shown in Figure 2e. It can be seen that although the three known DNA binding sites formed with the involvement of the L1-, L2-loops, and the α1-helix of H2A form some atom–atom contacts with DNA, the N- and C-terminal tails contribute substantially to the interactions with the nucleosomal DNA. The N- and C-terminal tails together on average are responsible for around 130 atom–atom contacts, which constitutes more than 60% of the overall number of H2A-DNA atom–atom contacts.

### 2.2. H2A C-Terminal Tail Interaction Patterns with the DNA Differ Between Histone Variants

**Figure 3:**
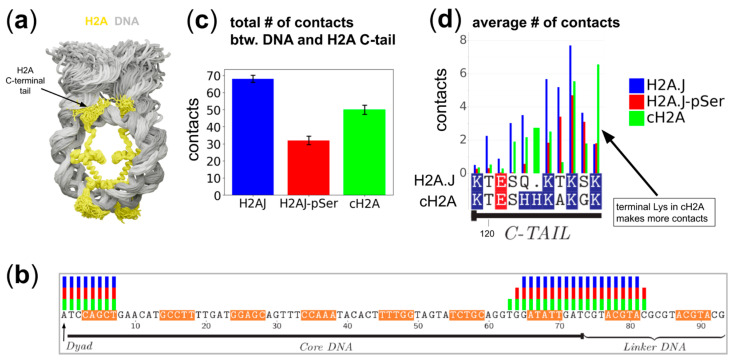
The contact profiles between H2A histones and DNA for different systems: (**a**) An overlay of snapshots showing only DNA and H2A histones for the H2A.J-containing system (N_H2A.J_); (**b**) Sites on DNA that were accessible for contacts with H2A C-terminal tails in different systems. The presence of a bar of a particular color indicates that the particular nucleotide participated in at least one contact in the respective simulation. The profile was symmetrized by taking into account contacts on both symmetry-related sides of the nucleosome. The DNA sequence on the *X*-axis starts from the nucleosome center (dyad) and runs towards the linker DNA ends. The segments where the DNA minor groove is facing towards the octamer are highlighted in orange (according to the analysis carried out in [46]); (**c**) The average number of contacts between H2A C-tails and the DNA for different simulated systems; (**d**) A comparative plot of the average number of atom–atom contacts for the three simulated systems plotted for the C-terminal tail of H2A histones. Positively and negatively charged residues are highlighted in blue and red, respectively.

**Figure 4:**
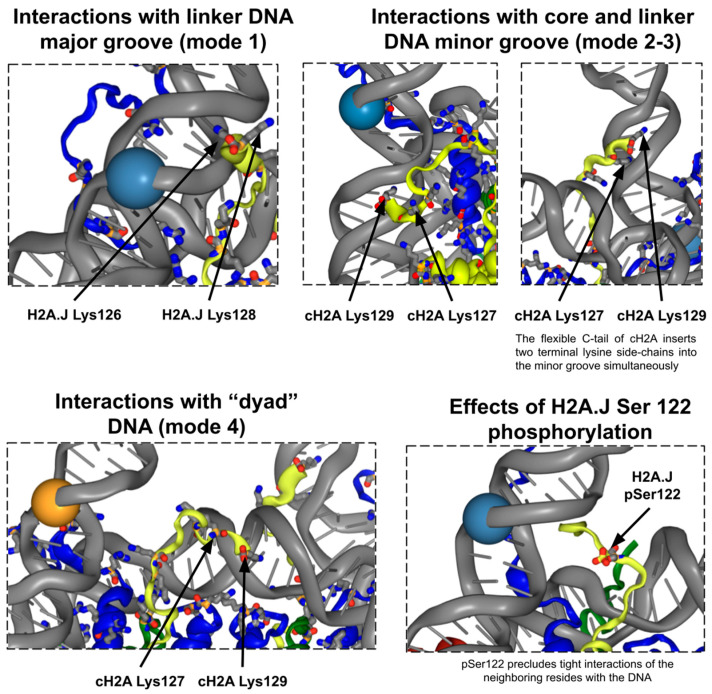
Different patterns of interactions between C-terminal tail residues and DNA. Orange or blue spheres show the border between nucleosomal and linker DNA segments. Histones H2A, H2B, H4 and DNA are colored in yellow, blue, green and gray, respectively.

To explore the details of H2A C-terminal tail dynamics and interaction patterns, we performed visual analysis and evaluated the contact patterns and hydrogen bond patterns for the three simulated systems (Figure 3, Supplementary Figures S5–S7, Supplementary Table S2) throughout the MD trajectory (see Methods). As seen in Figure 3b, the C-tails of H2A histones make contacts with the DNA in two locations: near the nucleosome dyad (up to 7–8 bp from the dyad) and near the nucleosomal DNA entry–exit site. In the latter case, the contacts are made with a region that spans up to one helical turn (~10 bp) of the nucleosomal DNA (located inside the nucleosome core particle) and up to one helical turn of the linker DNA segment (see representative snapshots in Figure 4). All three systems were able to contact these DNA locations; however, the number of contacts for the H2A.J_pSer_ system was substantially lower, consistent with the repulsion of phosphoserine from the DNA (Figure 3c,d). Although the microsecond timescale may not be enough to obtain a comprehensive sampling of histone tail conformations, there were also indications that the H2A.J C-tail makes more contacts with DNA than that of canonical H2A. This average contact pattern between DNA and the C-tail comes from a dynamic conformational switching of the tail between at least four modes of conformations (Figure 4): (1) the orientation toward the linker DNA segment when the end is lodged into the nearest major grove; (2) the extended conformation along the linker DNA when the end of the tail reaches the minor groove, with the side chains of terminal lysines often inserted into it; (3) the conformation when the H2A C-tail is folded back and interacts with the nearest minor groove of the DNA that is bound to the histone octamer; and (4) the orientation towards the dyad (see also Supplementary Figure S5b).

The comparative analysis of contacts and hydrogen bonding patterns between the simulated systems revealed that substitutions that introduced additional hydroxyl groups (A126 to T125 and G128 to S127) resulted in the formation of new hydrogen bonds with DNA and neighboring C-terminal tail residues (Supplementary Figure S6). While histone tail dynamics are known to require long simulation times to obtain reliable conformation sampling in MD simulations [37,47], our simulations at the microsecond timescale were suggestive of the following H2A.J-specific effects: (1) The phosphorylation of H2A.J-Ser122 had the most profound effect; it almost abolished the direct interactions between the H2A.J C-tail and the DNA in the region H2A.J-Thr120:Gln123 and greatly reduced interactions with the DNA of H2A.J-Lys124 (Figure 3d), although the downstream region H2A.J-Thr125-Ser127 was still able to maintain substantial interactions with the DNA. (2) The interactions with the DNA of the terminal lysine residue are decreased in systems containing H2A.J as compared to canonical H2A. While the first effect is explained by the repulsion between the phosphate groups of DNA and H2A.J-pSer122, the second effect likely stems from the decreased flexibility of the very end of the C-tail in H2A.J. Indeed, the flexible glycine hinge between the two lysine residues provided by H2A-Gly128 is replaced by H2A.J-Ser127 in H2A.J. The dihedral angle analysis of the dynamics of this terminal motif using Ramachandran plots confirmed a higher flexibility for the canonical H2A C-tail, sampling various dihedral conformations that were not accessible to the H2A.J system (Supplementary Figure S7). The presence of this flexible glycine allows canonical H2A to be able to simultaneously insert H2A-Lys127 and H2A-Lys129 side chains into one DNA minor groove, while such events were rarely observed for H2A.J. As illustrated in Figure 3d, the terminal KGK motif at the end of canonical H2A is able to make more stable contacts with the DNA than the terminal KSK motif of H2A.J. The observation of stable atom–atom contact formation for H2A.J-Lys124 and H2A.J-Thr125 suggests alternative interaction patterns employed by the H2A.J C-tail.

Taken together, our simulations demonstrate that canonical H2A-, H2A.J-, and H2A.J_pSer_-containing nucleosomes show different dynamical patterns of H2A C-tail interactions with the DNA.

### 2.3. The Effect of Ser40Ala Substitution on L1-Loop Conformation and in Intra- and Inter-Dimer Interactions Within the Nucleosome

H2A.J harbors alanine at position 40, as do many other canonical H2A isoforms, while certain have serine at position 40 (see Figure 1b). Our proteomics analysis suggests that around 20% of canonical H2A protein molecules present in the cell have serine at position 40 (Figure 1c). Since it was reported experimentally that S40A substitution makes nucleosomes more thermally stable [18], we focused our attention on analyzing the dynamical effects of this substitution. S40A substitution is located in the L1-loop region near the “anchor” arginine (R42), which forms stable contacts with the DNA minor groove at the superhelix location ±4 (four DNA helix turns away from the nucleosome center) [37]. The L1-loops of the two H2A histone copies inside the nucleosome are known to interact, stabilizing the overall nucleosome structure [3].

**Figure 5:**
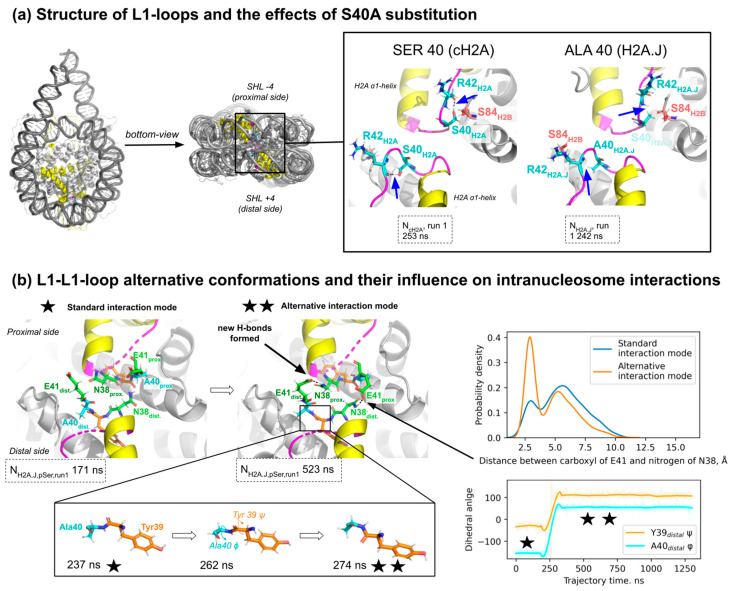
Molecular details of the effects of H2A/H2A.J S40A substitution on L1-loop conformation and dynamics: (**a**) The interactions within the H2A-H2B-dimer that are altered due to S40A substitution. H2A/H2A.J histones are colored in yellow, H2A/H2A.J L1-loop is shown in magenta. Blue arrows mark the presence of a hydrogen bond between S40 and R42 or the absence of this bond between A40 and R42; (**b**) Left: observed H2A.J L1-loop reorganization accompanied by a change in torsion angles of the peptide backbone between residues Y39 and A40 (see inset in a black frame) and formation of inter-H2A-H2B-dimer hydrogen bonds between E41 and N38. Right: the changes in the probability of distribution of the distance between donor and acceptor atoms of E41 and N38 residues (top) and changes in peptide backbone dihedral angles (bottom) upon L1-loop reorganization. One and two asterisks denote standard and alternative conformation modes of L1-loops, respectively.

Hydrogen bond analysis showed that during MD simulations, the H2A S40 side chain hydroxyl group makes hydrogen bonds with the backbone oxygen atom of R42 and occasionally with the backbone oxygen of H2B S84 (Figure 5a). Such contacts were present in 76% and 29% of N_cH2A_ MD trajectory frames, respectively. The presence of the S40-R42 hydrogen bond supported the bent conformation of the L1-loop as seen in X-ray structures of nucleosome core particles. H2A.J histone has an S40A substitution, which leads to a complete disappearance of the discussed hydrogen bond with either H2A.J R42 or H2B S84 due to the absence of the hydroxyl group in alanine (Figure 5a).

Despite the loss of the hydrogen bond upon S40A substitution, the overall structure of L1-loops in most MD simulations remained similar (Supplementary Figure S8a,b). However, in one simulation of the H2A.J-containing nucleosome (N_H2A.J, pSer_, run 1 simulation), the two torsion angles of the peptide backbone near the S40A substitution spontaneously rotated (Figure 5b). This was accompanied by an increase in the distance between A40 and the backbone of R42, so that if A40S substitution were to happen in this conformation, no hydrogen bond with R42 could be formed (Supplementary Figure S8c). Additional control MD simulations of H2A-H2B and H2A.J-H2B dimers without DNA also showed such reorganization of the H2A.J L1-loop (Supplementary Figure S9).

This alternative conformation of the L1-loop was correlated with the altered probability of contacts between the two H2A-H2B dimers in the nucleosome, particularly the probability of a hydrogen bond formation between H2A E41 on one half of the nucleosome and H2A N38 on the other side of the nucleosome (see Figure 5b, Supplementary Figure S8d). The residence time analysis of this hydrogen bond also suggested that the altered L1-loop conformation supported longer residence times of the said hydrogen bond (see Supplementary Figure S8e).

Taken together, the S40A substitution in H2A histones may lead to the altered conformation of L1-loops, likely resulting from the loss of a hydrogen bond between S40 and R42, which in turn alters the interaction patterns between the two H2A-H2B dimers in the nucleosome.

### 2.4. The Role of Ala10Val Substitution

**Figure 6:**
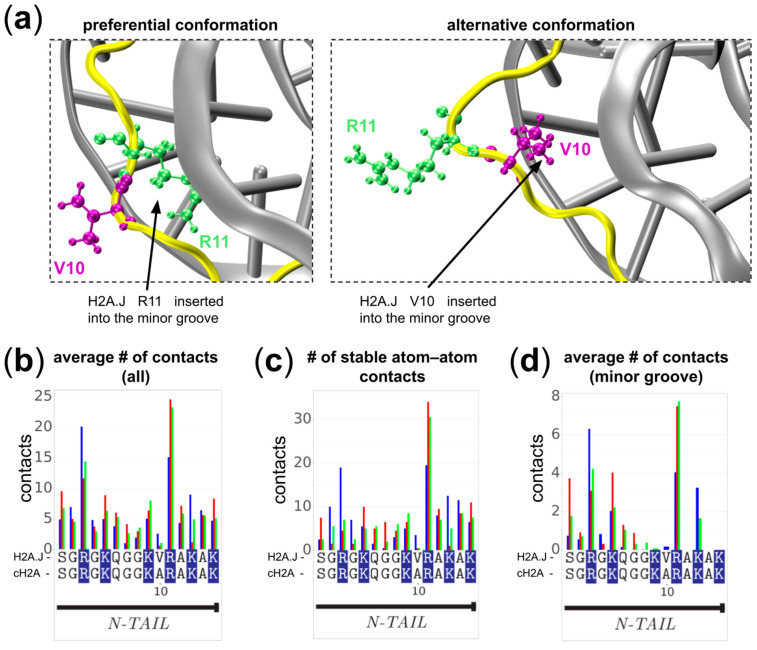
Dynamics of the H2A.J N-terminal tail (yellow) in the vicinity of H2A.J-Val10 substitution: (**a**) Snapshots showing the location of H2A.J Val10 and modes of interaction of the N-terminal H2A.J tail with the DNA minor groove. (**b**) A comparative plot of the average number of atom–atom contacts for the three simulated systems plotted for the N-terminal tail of H2A histones. (**c**) Same as (**b**), but the number of stable atom–atom contacts is shown (a stable atom–atom contact is defined here as a contact that was present in at least 25% of trajectory frames; the values are averaged over two copies of H2A in the nucleosome). (**d**) Same as (**b**), but contacts with the nucleobases’ atoms in the DNA minor groove are used to make the profile. Positively charged residues are highlighted in blue.

The Ala10Val substitution is one of the few strictly specific to H2A.J; it is located in the N-terminal tail of H2A (Figure 1b). Our analysis of the dynamics in the vicinity of H2A.J V10 showed that the N-terminal tail is much more stably associated with the DNA than the C-tail. It contributes to around 45% of atom–atom contacts between DNA and H2A (Figure 6b,c). The R11 residue is most often inserted into the DNA minor groove and forms stable contacts there (Figure 6a,d, and cluster analysis in Supplementary Figure S10). In this conformation stabilized by the R11 insertion, V10 points away from the minor groove with its side chain exposed to the solvent. In some instances we have observed the reorientation of the R11 away from the minor groove; in this case, V10 pointed towards the DNA minor groove (Figure 6a, left). The comparative analysis of contact profiles between different simulated systems did not reveal any statistically significant differences in DNA contact profiles that could be associated with A10V substitution.

## 3. Discussion

MD simulations are becoming an important tool to analyze nucleosome dynamics and its functional implications [34]. Here we have used this approach to characterize the dynamics of human nucleosomes containing H2A.J histone variants and their difference from canonical nucleosomes at the microsecond timescale. H2A.J histone is sequence-wise very similar to canonical H2A (around 94% identical), but harbors amino acid substitutions at specific locations. These differences apparently form the molecular basis for H2A.J-specific roles in cell senescence, inflammation, cancer, etc. However, previous X-ray structural analysis of H2A.J-containing nucleosome core particles (NCPs) did not reveal any substantial changes to the NCP structure [18]. In MD simulations, we have added linker DNA segments to the NCP, which are known to be important interaction partners for H2A C-terminal tails that vary between H2A.J and canonical H2As [48,49]. H2A C-tails have also been implicated in interactions with H1 histone, whose binding requires linker DNA segments [49]. Overall, this allowed us to explore specific dynamical properties of H2A.J nucleosomes in comparison to canonical ones and to the one phosphorylated at H2A.J-pSer122 (an H2A.J-specific phosphorylation site). For the latter end, we have developed specific modifications to the AMBER ff14SB force field. While the overall dynamics of the simulated nucleosomes were very similar, local changes in the dynamics and interaction patterns in the two regions were detected and analyzed: the C-terminal tail and the L1-loop of H2A.J.

According to our simulations, the C-terminal tail of H2A is quite dynamic and may form interactions with both linker and nucleosomal DNA segments. On the contrary, the N-tail manifests much less conformational dynamics. C-tails of different H2A variants have been shown to affect nucleosome structure and dynamics (e.g., H2A variants with short C-tails, such as H2A.B, H2A.P, and H2A.L are known to form nucleosomes with unwrapped DNA [28,50]), play important roles as sites of additional post-translational modifications (e.g., H2A.X S139 is a known phosphorylation site activated due to DNA damage response [51], H2A.Z.1 K101me2 contributes to the development of breast cancer [52], or interaction partners with other chromatin proteins (e.g., H1 [49,53]. The role of the H2A.J C-tail has not been extensively experimentally studied. Tanaka et al. showed that the substitution of the H2A.J C-tail to that of canonical H2A did not affect the thermal stability of nucleosomes [18]. They also showed that H2A.J nucleosomes were slightly more resistant to MNase digestions; however, whether this was due to the effects of C-tail or L1-loop A40S substitution is unclear. Data from Mangelinck et al. on co-immunoprecipitation of H2A and H1 histones suggested that H2A.J interaction with H1 histones is weaker than that of canonical H2As [17]. However, no in vitro biophysical studies of this interaction and implication of the H2A.J C-terminal tail have been reported. For canonical H2A, it has been shown both in cells and in biophysical experiments that truncation of its C-tail makes nucleosomes less stable and lowers their interaction strength with H1 histones [49]. Hence, a plausible hypothesis explaining the effects of H2A.J on H1 binding might include either direct interactions of the C-terminal tail of H2A.J with H1 or indirect competition of H2A.J with H1 for their binding to nucleosomal DNA.

The H2A.J C-tail is different from that of canonical H2As in at least four positions. The histidine-histidine dipeptide motif in H2A is replaced by a single glutamine (position 123) in H2A.J. This introduces an SQ-motif that may be phosphorylated similar to the well-known SQEY motif at the end of the H2A.X histone. The importance of H2A.J SQ-motif phosphorylation still remains to be studied, since a phosphorylation level of only up to 1% of H2A.J has been reported in mice after substantial irradiation [17]. In our simulations, the phosphorylation of H2A.J S122 resulted in a marked reduction in atom–atom contacts between the H2A.J C-tail and DNA. S122 is located next to E121, which is already negatively charged. Thus, phosphorylation creates a compact site of negative charge (of −3 electron charges), which undergoes electrostatic repulsion from the DNA. The contacts with the DNA in the vicinity of this site are thus sufficiently subdued. At the same time, pSer122 electrostatic attraction with neighboring positively charged lysines, disturbing the bonding of other tail’s residues with DNA, and providing an additional acceptor phosphate group may promote the formation of new intra-tail hydrogen bonds, changing tail conformation. All of this leads to weaker tail–DNA binding and potentially to the increase in linker DNA dynamics and reduced nucleosome stability. This effect may be similar to the phosphorylated C-terminal tails of H2A.X previously experimentally demonstrated [54]. Interestingly, the three lysine residues at the end of the H2A.J C-tail were still able to interact with DNA and form contacts in our simulations, even for the H2A.J-pSer122-containing nucleosomes.

The other difference between major canonical H2A isoforms and H2A.J are the changes in the residues that separate the three lysine residues at the C-end from each other: the KAKGK motif in canonical H2A is replaced by KTKSK in H2A.J. The H2A.J C-terminus is thus more hydrophilic, and our simulations show that it can form additional hydrogen bonds with the DNA as well as, on average, form more contacts with the DNA than the C-tail of canonical H2A. Following the addition of new donor and acceptor groups, all the C-terminal tail H2A.J-specific substitutions also demonstrate enhanced hydrogen bonding with other residues of the same tail. The additional hydrogen bonding interaction of H2A.J C-terminal tails with the linker DNA segments may be responsible for the stabilization of the nucleosomal DNA and moderation of DNA unwrapping, which is consistent with experimental observation of a slightly higher resistance of H2A.J nucleosomes to MNase digestion [18]. We also observed the difference in the internal dynamics and the flexibility of the canonical H2A tail. The presence of a flexible glycine hinge in canonical H2A allows the neighboring lysine to be more independent in their interactions with the DNA and together form more interaction with the DNA. It has been shown in experimental and computational studies that interactions of histone tails with nucleosomal and linker DNA modulate the possibility of other chromatin proteins interacting with histones and DNA in the nucleosomes [41,55,56]. We hypothesize that different dynamical interaction patterns of H2A C-tails with DNA demonstrated in our simulations may have similar implications. According to PhosphositePlus, the three terminal lysine residues in canonical H2A (K118, K119, K125) and H2A.J K124 may be ubiquitinated [57]. Ubiquitination is an important post-translational modification of histones. For instance, H2AK119 is an important ubiquitination site involved in the formation of heterochromatin through a Polycomb group protein-mediated mechanism, which is located in the H2A C-tail [58]. Given the observations of Mangelinck et al., who showed that ectopic expression of H2A.J stimulates the expression of repetitive DNA elements, it is tempting to speculate that H2A.J may influence the Polycomb-mediated suppression of repetitive DNA, which is known to occupy around two-thirds of human genomes [59].

Our MD simulations suggested that H2A L1-loop dynamics of nucleosomes were altered through S40A substitution. It is important to note that this is not an exclusively H2A.J-specific substitution since some of the canonical H2A isoforms also harbor alanine at this position. The L1-loops of the two copies of H2A histones interact in nucleosomes, forming an important stabilizing interaction. The structure of the loops is known to differ in certain variants, like H2A.Z and macroH2A (reviewed in [28]). Interestingly, only one substitution in the L1-loop may change its structure and be responsible for a more rapid exchange of the histone in the cell, as exemplified by the human H2A.Z.1- and H2A.Z.2-containing nucleosomes [30]. In our simulations, we observed that the altered conformation of L1-loop in H2A.J nucleosomes increased the probability of additional hydrogen bonds’ formation between the two H2A.J/H2B dimers inside nucleosomes. We hypothesize that this may be one of the factors explaining the higher thermal stability of H2A.J-nucleosomes compared to canonical nucleosomes [18]. It is currently unclear to what extent this increased thermal stability may be functionally important; however, there are well-known examples when thermostability changes due to point substitutions in H2A are functionally important. Particularly, M51L substitution found in certain canonical H2A isoforms decreases the H2A-H2B melting temperature by around 3 °C, and when such isoforms are exogenously overexpressed, cell proliferation is promoted in a context-dependent manner, apparently by loosening the chromatin structure [12,60].

The A10V substitution is specific to H2A.J. Experimental mutational studies have suggested that it moderates the effects of H2A.J in promoting transcriptional activity, likely by recruiting another factor [17]. The neighboring H2AK9 is a site that may be acetylated, methylated, biotinylated, succinylated, benzoylated, 2-hydroxyisobutyrylated, and hydroxybutyrylated [61]. Another substitution in H2A.J is T16S, which is not variant-specific and is present in the majority of cH2A isoforms. The substitution is localized near the well-known monoubiquitination site H2AK15ub, which contributes to non-homologous end-joining DNA repair [61]. The context of the PTM site is well known to affect the activity of the enzymes targeting it [62]. In our simulations, we show that this site is in tight contact with the DNA, which may additionally affect and regulate its availability for trans-acting factors.

Taken together, our study has revealed certain unique dynamical features of nucleosomes containing the H2A.J histone variant and its phosphorylated form. We have characterized how H2A.J-specific sequence features affect (1) the interaction patterns of the H2A.J C-terminal tail with the DNA near its entry/exit into the nucleosome core, (2) the interactions between the two H2A-H2B dimers inside nucleosomes at the L1-L1-loop site, and (3) the configuration of post-translationally modified sites located in the N-terminal tail of H2A. In the future, additional experimental studies such as NMR relaxation experiments for tails’ dynamics [63] as well as thermostability or salt concentration disassembly experiments with FRET labels on H2A-H2B dimers [64] could strengthen our conclusions. Our results contribute to the further understanding of the molecular mechanisms behind functional implications conferred by the deposition of H2A.J histones into nucleosomes along the genome. Particularly, we hypothesize that the different dynamics and interaction patterns of H2A.J C- and N-terminal tails might affect their ability to undergo post-translational modification and recruit downstream chromatin effectors.

Our study is not without limitations. Particularly, our MD analysis was limited by the timescale of several microseconds, and thus the sampling of conformations was limited. It is likely that at longer timescales and with more extensive conformational sampling, more statistically stringent characterization of the different interaction modes of the flexible histone tails and their effects on DNA unwrapping can be made. The refinement of force fields in order to better model disordered proteins and protein DNA interactions is an ongoing effort [43,65,66]. The use of the next generation of force fields might improve the predictive power of MD simulation studies of nucleosomes, especially with respect to modeling the conformation of disordered histone tails.

## 4. Materials and Methods

### 4.1. Building Nucleosome Systems for MD Simulations

In this study, the initial model of a nucleosome with canonical H2A (N_H2A_) was built using human nucleosome structure (PDB ID 5AV9) that included isoform 2 of human canonical H2A histone (encoded by H2AC4 or H2AC8 genes) [67]. Full-sized histone terminal tails and linker DNA segments 20 bp in length were added using UCSF Chimera [68] and 3DNA [69] software, respectively. The linker DNA segments were essential as the sites of potential interactions for histone tails. The canonical nucleosome was used to derive the nucleosome model harboring the H2A.J histone variant (N_H2A.J_) by substituting the corresponding residues in the H2A histone using UCSF Chimera. Analogously, a phosphate group was added to H2A.J Ser122 to simulate the effects of phosphorylation (N_H2A.J-pS122_). The protonation states of residues were assessed with the PROPKA software [70], and all histidines were modeled in non-protonated states (neutral charge of their sidechain). H2A-H2B and H2A.J-H2B dimer systems were extracted from the corresponding nucleosome models, and for these models, disordered histone tails were truncated (16–102 and 34–122 residues of segments C and D were taken, respectively). Constructed models were solvated in a rhombododecahedral cell with periodic boundary conditions and minimal distance from nucleosome to the cell boundary of 2 nm using the TIP3P explicit water model [71] with a 150 mM NaCl concentration. After the first short MD simulations needed for histone terminal tail condensation onto the DNA were run, the models were resolvated in smaller rectangular boxes (20 × 18 × 30 nm) under the same conditions.

### 4.2. Simulation Parameters

Simulation protocols followed those used previously [37,72,73]. Briefly, all simulations were carried out with the GROMACS 2020.2 software package [74]. The AMBER ff14SB force field [75] with parmbsc1 DNA parameter correction [76] and CUFIX ion parameter correction [65] were used. The force field parameters were also appended by adding newly designed parameters for phosphoserine amino acid residue (see below Section 2.4). To avoid potential fraying of terminal DNA base pairs during simulations, a harmonic potential with a force constant of 1000 kJ mol^−1^ nm^−2^ was applied to the distance between glycosidic nitrogen atoms of the terminal base pairs. The prepared systems were energy minimized using the steepest descent gradient method with positional restraints on heavy atoms of 500 kJ mol^−1^ nm^−2^. Then the five stages of equilibration with a gradual relaxation of harmonic positional restraints imposed on all heavy atoms were performed: 1. 100 ps with positional restraints of 500 kJ mol^−1^ nm^−2^ with a timestep of 0.5 fs; 2. 200 ps with positional restraints of 50 kJ mol^−1^ nm^−2^ with a timestep of 2 fs (further, the timestep was kept at 2 fs); 3. 200 ps with positional restraints of 5 kJ mol^−1^ nm^−2^; 4. 200 ps with positional restraints of 0.5 kJ mol^−1^ nm^−2^; 5. 200 ps free simulation. The following production run was performed with a 2 fs step; trajectory frames were saved every 1 ns. For all simulations, an NPT ensemble with a temperature of 300 K using the velocity rescale scheme [77] and a 1 bar pressure coupling using a Parrinello–Rahman barostat [78] were used. The simulations progressed at an average speed of 20 ns per day. All six resulting trajectories have a length of 1500 ns. Simulations were performed on the Lomonosov-2 supercomputer using 1 computing node, having 14 CPU cores and one NVidia Tesla K40 GPU [79] and a high-performance computer cluster using 1 computing node, having 40 CPU cores and one NVidia GeForce RTX 2080 Ti GPU. To prevent nucleosome diffusion in the simulation cell, C-α atoms of H3 histone globular domains (residue numbers 64–78, 86–114, 121–131) were slightly constrained with a harmonic restrain potential of 0.003 kcal/mol/A^2^.

### 4.3. Trajectory Analysis

Programs and pipelines for analysis followed those used by us previously [37,72,73] and included Python 3 scripts with the integration of GROMACS [74], MDAnalysis [80], VMD [81], and 3DNA [69]. The analysis of nucleosomal conformations was performed by projecting the positions of amino acid Cα-atoms and DNA base pair centers onto the planes of the nucleosome reference frame defined by the nucleosome superhelical axis and the dyad axis as described in [37]. The extent of DNA unwrapping from the histone octamer was counted as the number of nucleosomal DNA base pairs starting from the nucleosome entry–exit site (the site is located 73 bp away from the nucleosomal DNA center, the so-called dyad) that moved away from the positions of every base pair in the initial structure by more than 7 Å. Atom–atom contacts were calculated using MDAnalysis; only heavy atom contacts at a distance of less than 4 Å were considered. The statistical uncertainty for the average number of contacts was estimated as the standard deviation of the number of contacts divided by the square root of the number of independent observations (estimated as the trajectory length divided by the autocorrelation time of the said variable). Stable atom–atom residue-nucleotide contacts were defined as those where any atom of a given protein residue and any atom of a given nucleotide would be in contact in at least 25% of analyzed trajectory frames. Hydrogen bonds were counted and analyzed with the VMD HBond plugin or manually using MDAnalysis. The donor–acceptor (D-A) distance and D-H-A angle cutoff were set to 3 Å and 20 degrees, respectively, and both backbone and sidechain atoms of residues were considered. The distance between two residues was measured as a mean distance between pairs of non-hydrogen residues’ atoms.

### 4.4. Force Field Modification

To model the phosphorylated serine amino acid residues, we extended the AMBER ff14SB force field with parameters for the said residue. To this end, to calculate the atomic charges for the modified residue, we created a tripeptide system with phosphoserine (pSer) between the two alanines (Ala) using PyMOL [82] and its plugin PyTMs [83]. PsiRESP [84] was then used to calculate the atomic charges of tripeptide. The following constraints were employed during charge calculation: an overall charge of −2 electron charges; the charges of the backbone atoms were constrained to their values in the AMBER ff14SB parameter set; and the charges of oxygen atoms in the phosphate group were set to be equal. The topology of the tripeptide was generated using ACPYPE [85]. The resulting pSer parameter set was integrated into the AMBER ff14SB force field for MD simulations. Detailed information about this parametrization and its validation can be found in Supplementary Methods. The force field with pSer parameters is accessible on GitHub at https://github.com/intbio/gromacs_ff/tree/master/amber14sb_parmbsc1_cufix_PTM.ff (accessed on 6 November 2024).

### 4.5. Histone Abundance Analysis Using Data from PaxDB

To obtain relative abundance of proteins representing different canonical histone isoforms and H2A.J, we relied on the proteomics data provided by the PaxDB comprehensive absolute protein abundance database in its “H.sapiens—Whole organism (Integrated)” dataset [86]. This dataset provides integrated data coming from many proteomics studies that have been normalized and combined. For the sequence alignment, the multiple alignment program MUSCLE was used [87].

## Data Availability

MD simulation trajectories and protocols are available for preview and can be downloaded from GitHub at https://github.com/intbio/Kosarim_et_al_2024/ (accessed on 6 November 2024).

## Supplementary Materials

The following supporting information can be downloaded at: https://www.mdpi.com/article/10.3390/ijms252212136/s1.
