# Supplementary material for: Molecular Dynamics Simulations of Nucleosomes Containing Histone Variant H2A.J
Source: Int J Mol Sci. 2024 Nov 12;25(22):12136. doi: 10.3390/ijms252212136 (PMC11595175; doi:10.3390/ijms252212136)
Supplement: Supplementary file 1 [file ijms-25-12136-s001.zip › ijms-3265176-supplementary.pdf]

SUPPLEMENTARY INFORMATION

Molecular dynamics simulations of nucleosomes containing histone variant H2A.J

Nikita A. Kosarim[1], Anastasiia S. Fedulova[1], Aleksandra S. Shariafetdinova[1], Grigoriy A. Armeev[1] and Alexey K. Shaytan[1,2]*

[1] Department of Biology, Lomonosov Moscow State University, 119234 Moscow, Russia
[2] Institute of Gene Biology, 119334 Moscow, Russia
* Correspondence: shaytan_ak@mail.bio.msu.ru ; Tel.: +7-(495)-939-5738

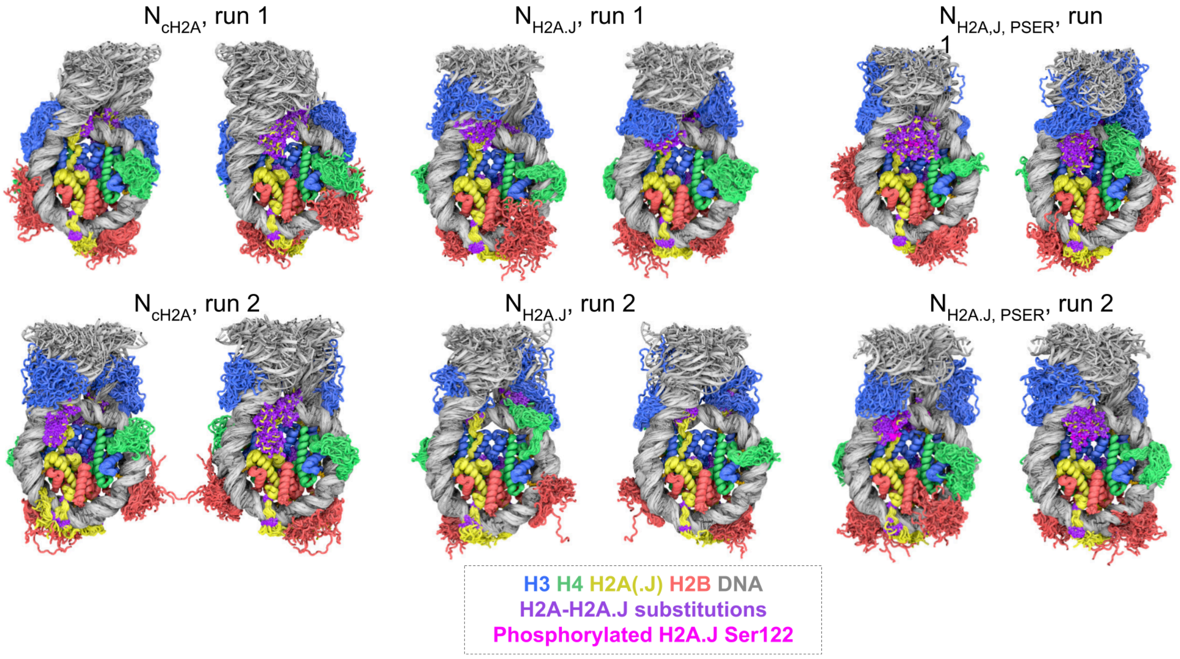

Supplementary Figure S1. MD simulations overview with overlaid snapshots. MD snapshots overlayed spaced 10 ns apart are shown. Interactive trajectory preview is available at https://intbio.org/Kosarim_et_al_2024/

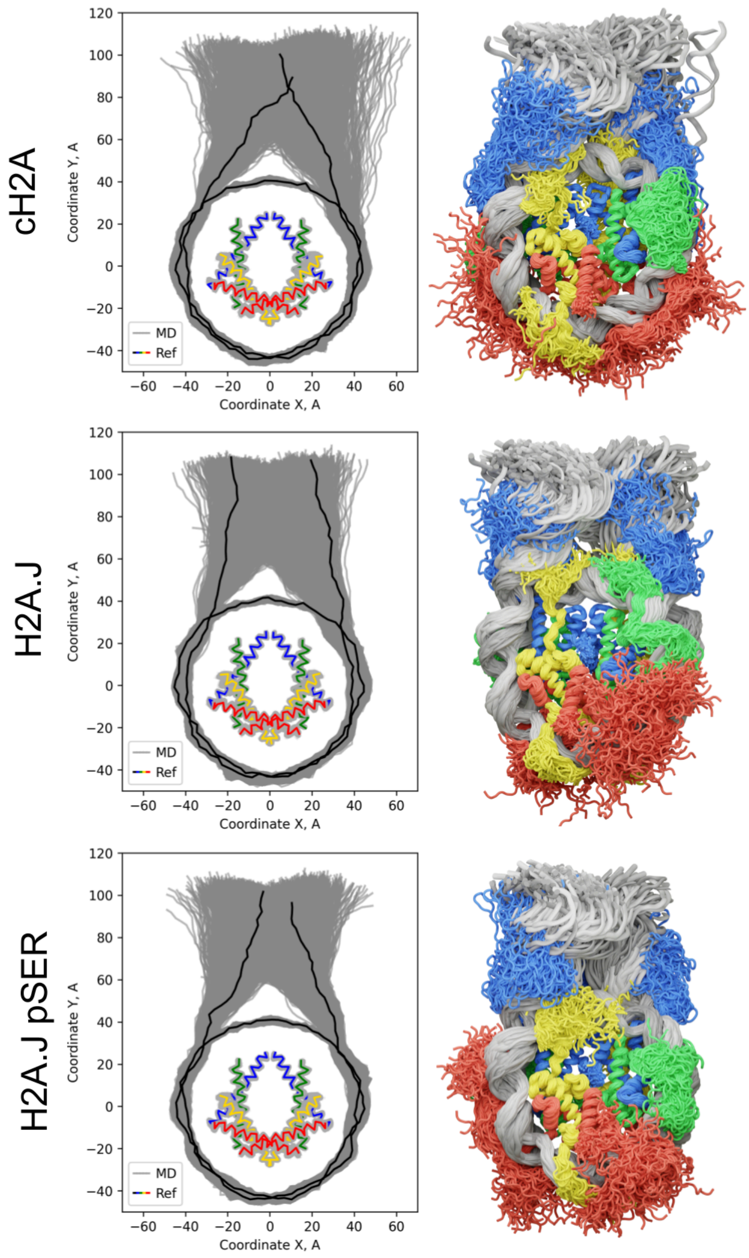

Supplementary Figure S2. MD simulations overview with overlaid projections. Same as Figure 2b in main text but for all simulated systems.

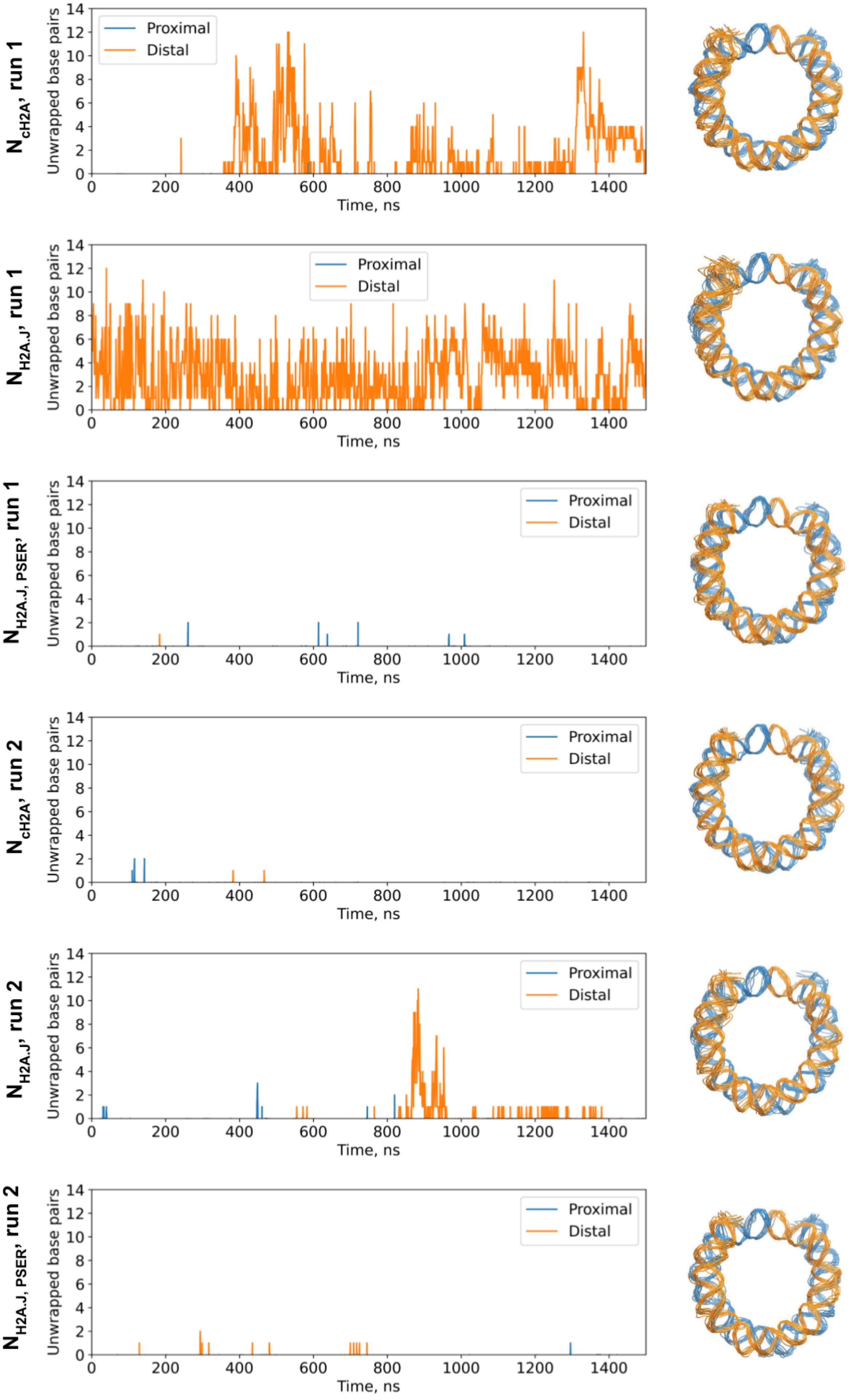

N_cH2A, run 1
N_H2A.J, run 1
N_H2A.J, PSER, run 1
N_cH2A, run 2
N_H2A.J, run 2
N_H2A.J, PSER, run 2
Unwrapped base pairs
Time, ns
Proximal
Distal

Supplementary Figure S3. Overview of DNA unwrapping in MD simulations. Nucleosome unwrapping profiles (left) and corresponding overlays of MD snapshots spaced 100 ns apart (right).

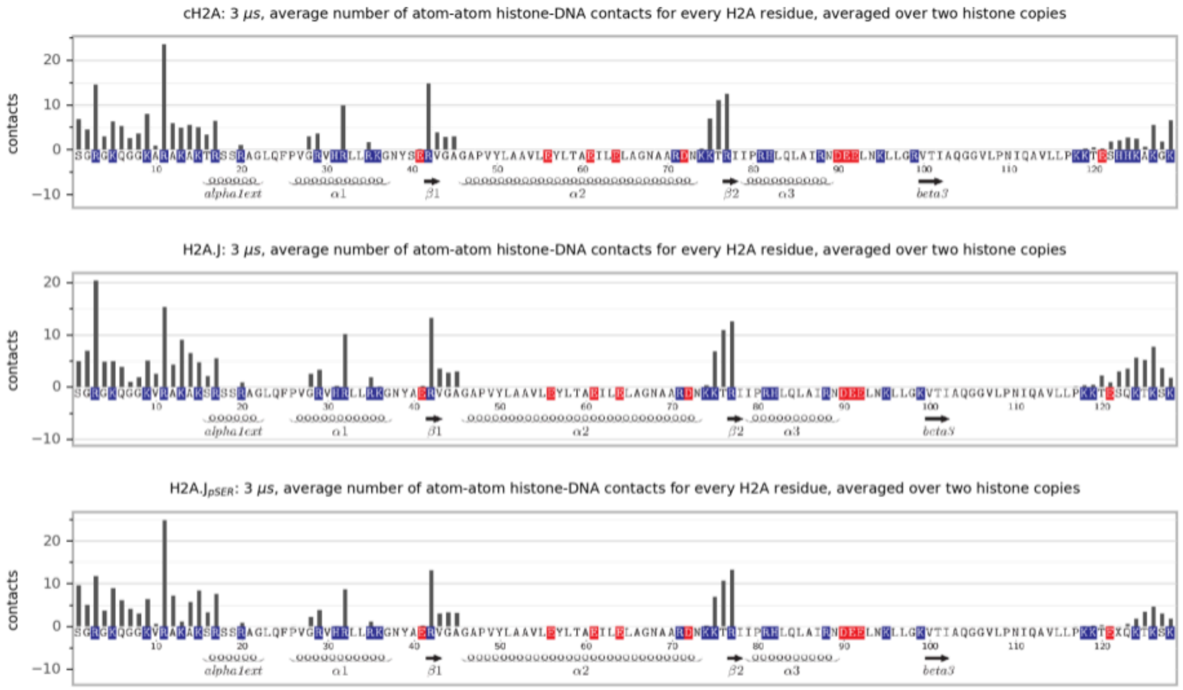

Supplementary Figure S4. **Histone-DNA contacts of H2A.** The profiles of the average number of atom-atom contacts between H2A histone residues and DNA for all systems.

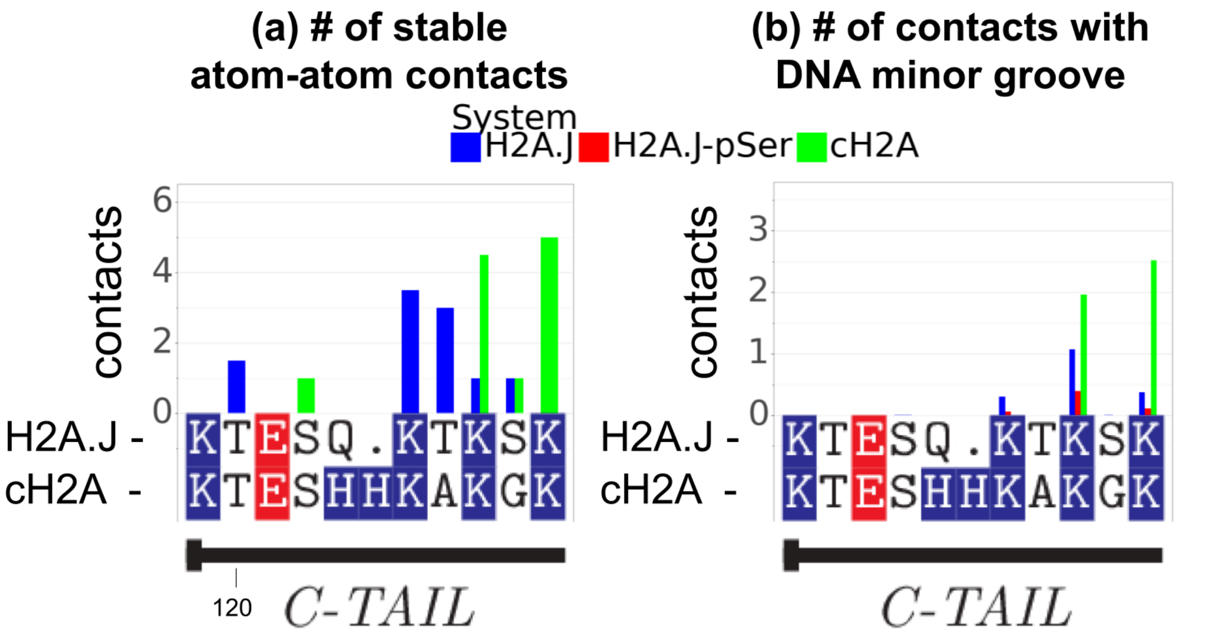

Supplementary Figure S5. **Histone-DNA contacts of H2A C-tail.** (a) Number of atom-atom stable contacts between H2A C-terminal tail and DNA for each simulated system. (b) H2A C-terminal tail interactions with the DNA bases atoms located in the minor groove.

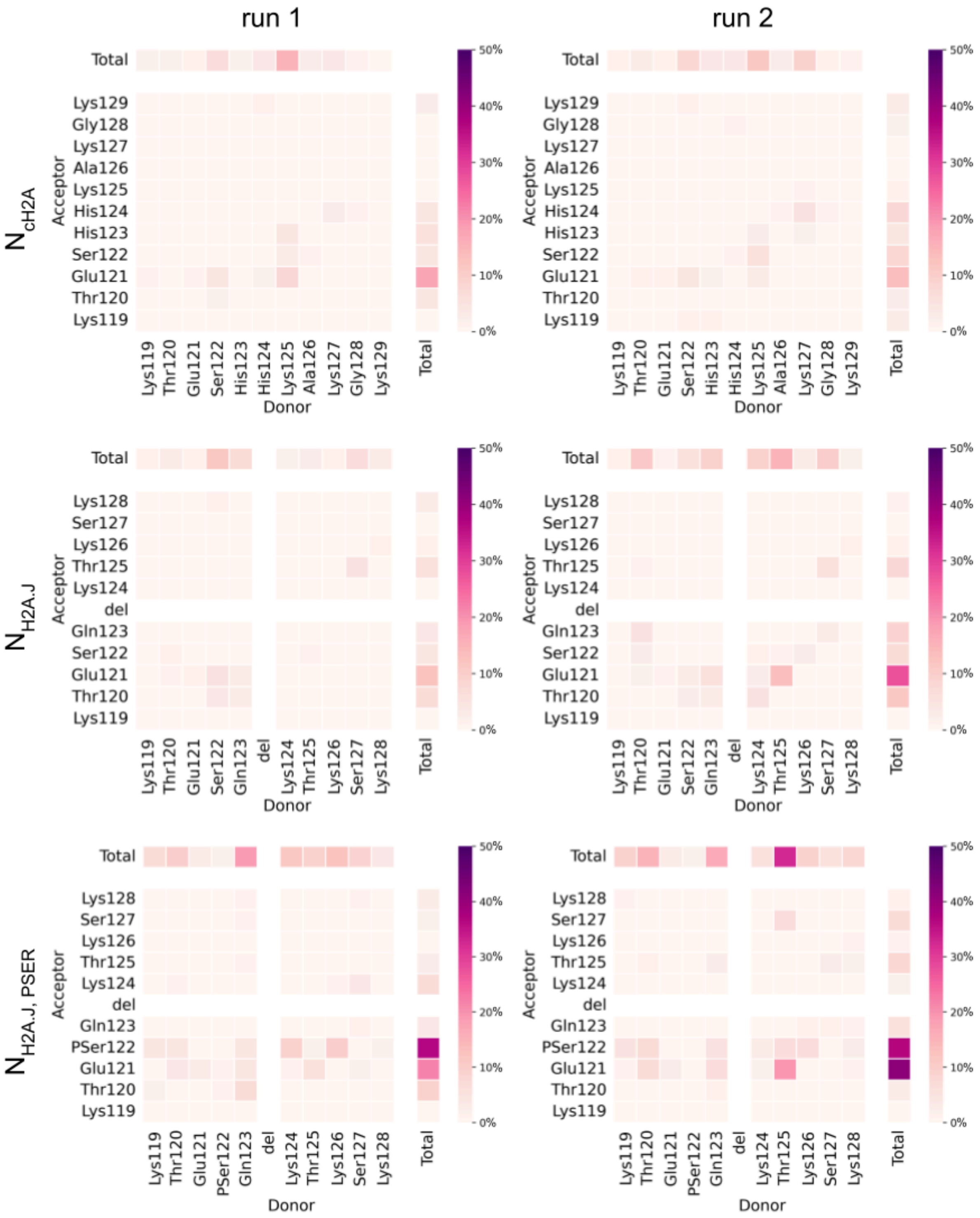

Supplementary Figure S6. Occupancy of hydrogen bonds between cH2A, H2A.J and H2A.J$_{pSer}$ C-terminal tail residues.

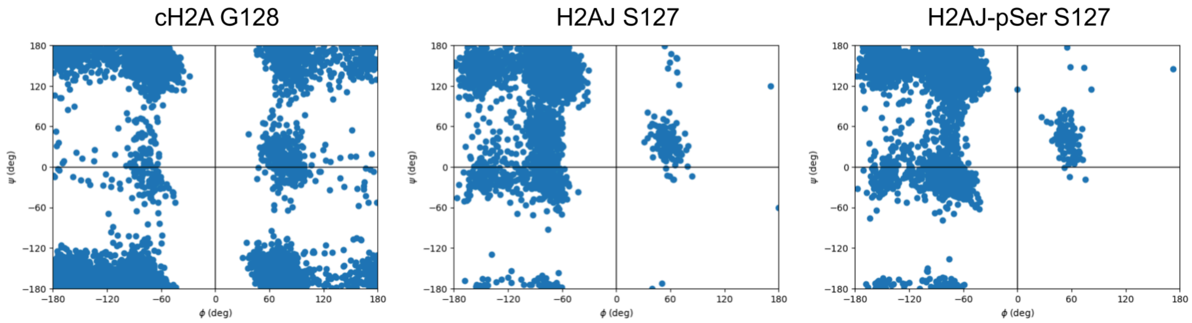

Supplementary Figure S7. Ramachandran maps of the backbone dihedral angles for canonical H2A G128 and H2A.J S127.

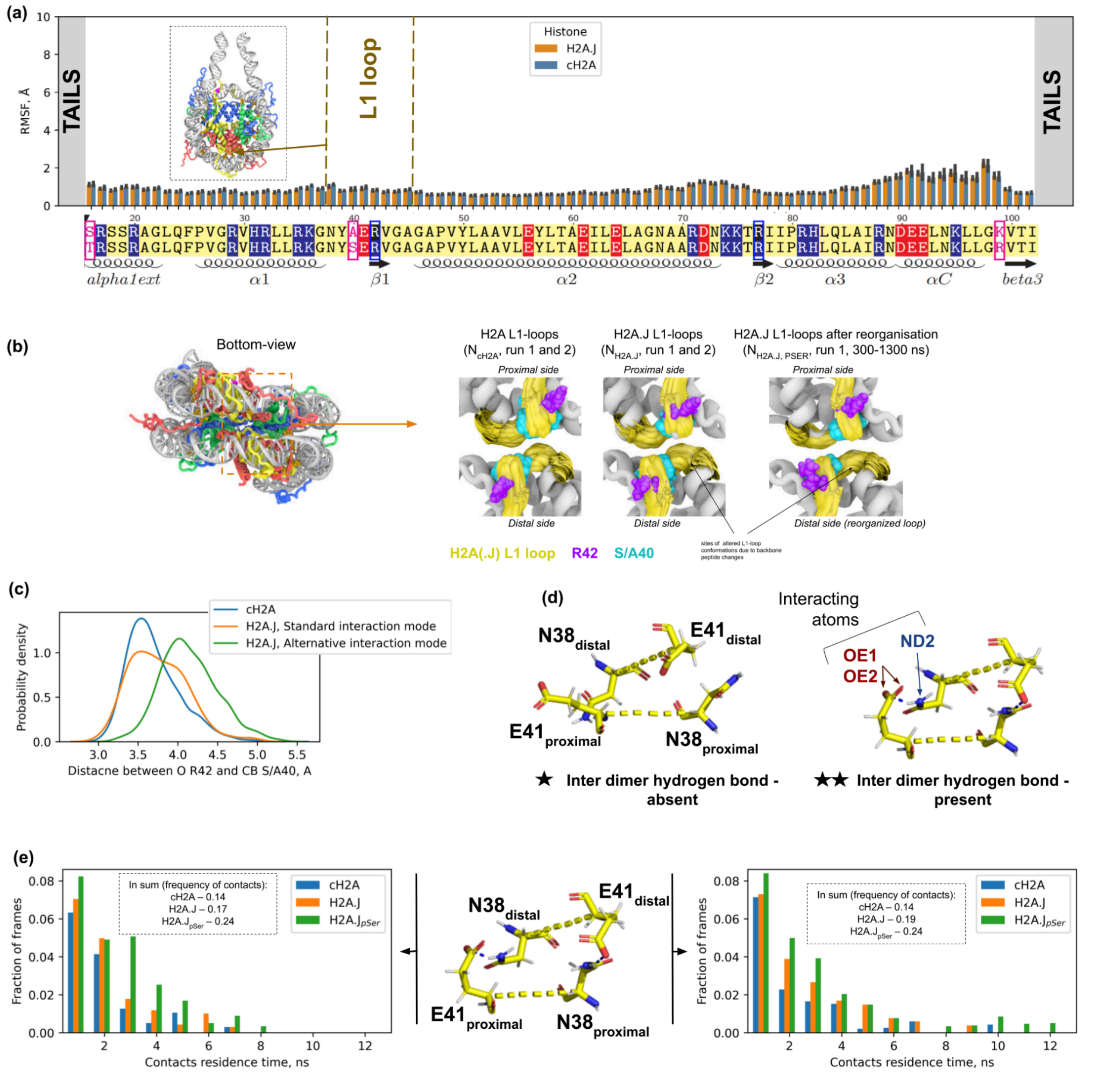

Supplementary Figure S8. Details of H2A L1-loop interactions upon S40A substitution. **(a)** RMSF profiles of Cα atom positions in the MD simulation of H2A- and H2A-J-containing nucleosomes. Only the globular region of H2A/H2A.J histones is shown. **(b)** H2A(.J) L1-loop conformations in MD simulations and its inter-(H2A/H2A.J-H2B)-dimer contacts. Zoom-up view of L1-loop regions. Overlay of MD frames. **(c)** Distance between Cβ-atom of S/A40

residue and O atom of R42 in MD simulations.**(d)** Two interacting residue pairs which constitute the inter-dimer contacts in less-effective (left) and more-effective (right) configuration. **(e)** Frequency of inter-dimer contacts and their residence times in MD simulations.

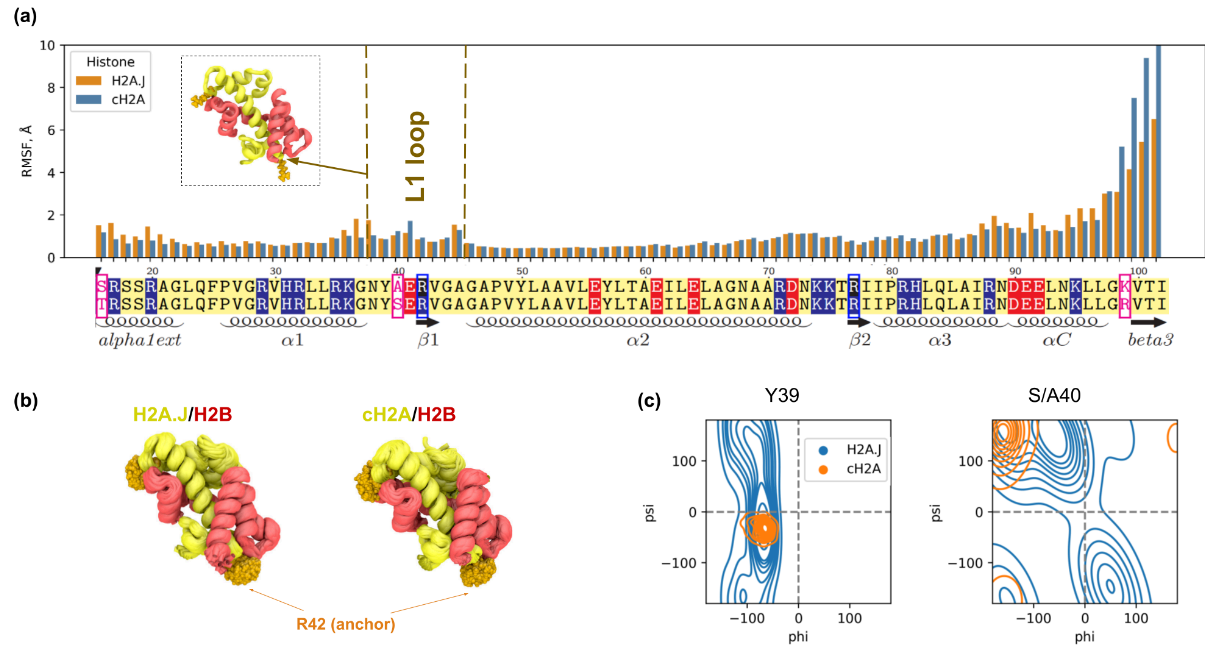

Supplementary Figure S9. MD simulation of histone dimers H2A-H2B and H2A.J-H2B with truncated histone tails. (a) RMSF profiles of Cα atom positions in the MD simulations. **(b)** Overview of the MD simulations is shown as overlayed MD frames. **(c)** Ramachandran angle values in the MD simulations for residues Y39 and S/A40.

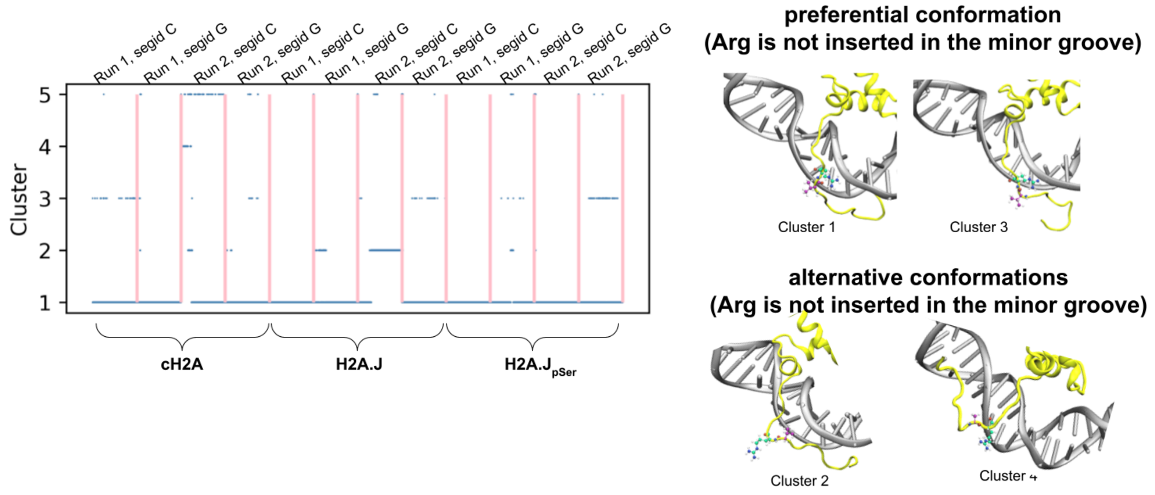

Supplementary Figure S10. Cluster analysis of the H2A N-tail conformation in MD simulations. Cluster analysis was performed using Gromacs with a cut-off of 0.35 nm. The analysis detected 12 clusters, the plot in the figure shows the first four clusters, and other smaller clusters are denoted as the 5th cluster. Pink lines denote each independent trajectory and each of two N-tail copies in the nucleosome. Representative states of each cluster are shown as snapshots of MD simulation frames.

Supplementary Tables

Supplementary Table S1. Details of the simulated systems.

Nucleosomes

System	N_{cH2A}, run 1	$N_{H2A.J}$, run 1	$N_{H2A.J,\ pSer}$, run 1	N_{cH2A}, run 2	$N_{H2A.J}$, run 2	$N_{H2A.J,\ pSer}$, run 2
Nucleosome atoms	27722	27704	27710	27722	27704	27710
Cation number	714	714	718	714	714	718
Anion number	488	488	488	488	488	488
Water	538380	538332	538281	538116	538179	538287
Trajectory length	1500	1500	1500	1500	1500	1500

Dimers

System	H2A-H2B	H2A.J-H2B
Dimer atoms	2844	2828
Cation number	73	72
Anion number	85	84
Water	80313	79047
Trajectory length	1500	1500

Supplementary Table S2. Occupancy of hydrogen bonds between C-terminal tails and DNA, %

Number	119	120	121	122	123	124	125	126	127	128	129
	Lys	Thr	Glu	Ser	His	His	Lys	Ala	Lys	Gly	Lys
N_{cH2A}. run 1	3,27	14,67	3,27	54,14	30,01	15,08	12,75	1,07	93,08	17,93	64,21
Tail 1	2,27	3,34	0,40	43,40	20,72	12,40	1,41	0,80	83,47	0,66	56,74
Tail 2	1,00%	11,33	2,87	10,74	9,29	2,68	11,34	0,27	9,61	17,27	7,47
N_{cH2A}. run 2	2,82	5,87	0,26	38,73	13,24	10,52	10,46	2,21	7,90	7,21	31,99
Tail 1	1,75	4,14	0,13	30,80	9,10	4,34	3,60	2,07	2,42	4,53	25,61
Tail 2	1,07	1,73	0,13	7,93	4,14	6,18	6,86	0,14	5,48	2,68	6,38
	Lys	Thr	Glu	Ser	Gln	-	Lys	Thr	Lys	Ser	Lys
$N_{H2A.J}$. run 1	2,79	35,74	1,54	36,28	28,40	-	34,41	77,92	65,55	58,62	14,20
Tail 1	0,79	5,00	1,27	33,14	20,60	-	15,08	58,59	36,61	45,88	11,68
Tail 2	2,00	30,74	0,27	3,14	7,80	-	19,33	19,33	28,94	12,74	2,52
$N_{H2A.J}$. run 2	3,34	48,53	14,47	71,21	37,89	-	8,67	71,80	62,42	63,95	14,22
Tail 1	0,20	41,33	14,00	22,66	8,82	-	4,79	41,73	52,41	51,80	10,34
Tail 2	3,14	7,20	0,47	48,55	29,07	-	3,88	30,07	10,01	12,15	3,88
	Lys	Thr	Glu	pSer	Gln	-	Lys	Thr	Lys	Ser	Lys
$N_{H2A.J.\ PSER}$. run 1	1,42	0,73	0	0	6,79	-	3,48	81,48	24,88	52,01	14,23
Tail 1	1,15	0,73	0	0	4,65	-	1,80	75,55	15,75	30,35	4,55
Tail 2	0,27	0	0	0	2,14	-	1,68	5,93	9,13	21,66	9,68
$N_{H2A.J.\ PSER}$. run 2	2,87	11,46	0,07	0	5,60	-	7,42	32,87	44,11	86,65	10,11
Tail 1	2,60	11,26	0,07	0	3,60	-	4,82	19,20	23,02	58,70	4,15
Tail 2	0,27	0,20	0	0	2,00	-	2,60	13,67	21,09	27,95	5,96

Supplementary methods

1. Calculation of restrained electrostatic potential atomic charges

We create a small peptide model system with phosphoserine (pSer) between two alanines (Ala) to determine the atomic charges for the AMBER ff14SB force field supplemented with parmbsc DNA and CUFIX ion parameter corrections. Tripeptide model with phosphoserine was made using PyMOL, especially its plugin PyTMs. PsiRESP was used to calculate atomic charges of tripeptide. We made the following constraints: an overall charge of -1, the charges of the backbone atoms were constrained to their values in the AMBER ff14SB parameter set and the charges of oxygen atoms in the phosphate group were made equivalent. The topology of tripeptide was generated using ACPYPE. The resulting pSer parameter set was integrated in AMBER ff14SB force field for further MD simulations. Force field with pSer parameters for further usage is accessible at https://github.com/intbio/gromacs_ff/tree/master/amber14sb_parmbsc1_cufix_PTM.ff

2. Testing of the pSer topology parameters by MD simulations

To test prepared pSer topology we performed MD simulations of tripeptides Ala-pSer-Ala and Ala-Ser-Ala (as a reference system). MD calculations for both tripeptide models were made with the AMBER14SB with parambsc1 and CUFIX force field and TIP3P water model. The charges of the systems were equated to -1 and 0 respectively by adding Na^+ and Cl^- ions. Minimizations of the solvated model peptides were started by relaxing the solvent molecules while the solute atoms were kept fixed. After 10.000 steps of solvent relaxation, a 100 ps of equilibration with positional restraints of 500 kJ/mol/A^2 (with 0.5 fs time step) and 200 ps of equilibration without restraints were performed. MD simulations were conducted at 300 K and 1 bar under NPT conditions using periodic boundary conditions and a time step of 2 fs. Simulations of tripeptide with phosphoserine and tripeptide with serine were carried out for 1084 ns and 2347 ns respectively. MD simulations show the stability of Ala-pSer-Ala tripeptide in the AMBER ff14SB force field. For more details, the analysis of dihedral angles was performed. From the Ramachandran plot (see Supplementary Methods Figure below) it can be proved that, according to the values of the dihedral angles for phosphoserine, this model does not lead to a change in protein folding.

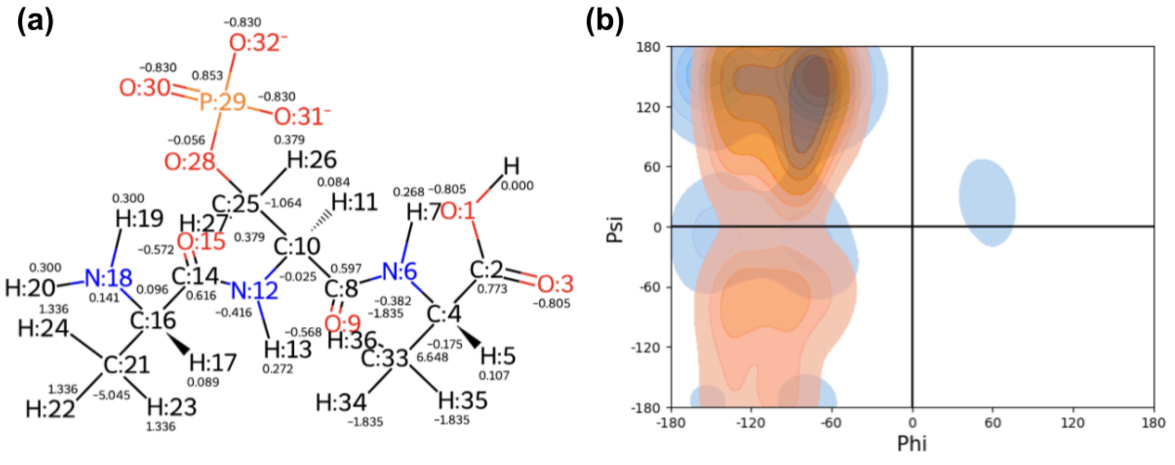

Supplementary Methods Figure. Details of phosphoserine parametrization. (a) The Ala-pSer-Ala tripeptide model with atomic charges. (b) Ramachandran plots of the Ser (blue) and pSer (orange) residues in MD simulations of Ala-Ser-Ala and Ala-pSer-Ala tripeptides.
